# Biallelic MCM10 Deficiency Presenting with Natural Killer Cell Deficiency, Poikiloderma and EBV-Associated Malignancy

**DOI:** 10.1007/s10875-026-02045-7

**Published:** 2026-07-30

**Authors:** Yael Hoffman, Alina Kurolap, Hila Ledermann Nahmias, Adi Mory, Tal Freund, Avraham Unterman, Uri Hamiel, Hagit Baris Feldman, Daphna Marom, David Hagin

**Affiliations:** 1 The Genetics Institute and Genomics Center, Tel Aviv Sourasky University Medical Center, 6 Weizmann St, Tel Aviv, 64239 Israel; 2 Allergy and Clinical Immunology Unit, Department of Medicine, Tel Aviv Sourasky University Medical Center, 6 Weizmann St, Tel Aviv, 64239 Israel; 3 Institute of Pulmonary Medicine, Center of Excellence for Interstitial Lung Diseases, Tel Aviv Sourasky University Medical Center, Tel Aviv, Israel; 4 Gray Faculty of Medical & Health Sciences, Tel Aviv University, Tel Aviv, Israel

**Keywords:** Inborn errors of immunity (IEI), Primary immunodeficiency disorders (PIDD), NK-cell deficiency (NKD), MCM10, Minichromosomopathy

## Abstract

**Purpose:**

Defects in components of the CDC45–MCM2-7–GINS (CMG) helicase complex have been associated with natural killer (NK) cell deficiency in rare inborn errors of immunity. Pathogenic biallelic variants in *MCM10* have been reported in a limited number of individuals with severe immunologic and developmental phenotypes. We aimed to further delineate the clinical and immunologic spectrum of *MCM10* deficiency.

**Methods:**

Singleton exome sequencing, RNA splicing analysis, flow cytometry–based immunophenotyping, telomere length assessment, radiation sensitivity testing, and in silico analysis of publicly available single-cell RNA sequencing datasets were performed.

**Results:**

A six-year-old boy presented with poikiloderma, photosensitivity, growth failure, and immune dysregulation. Exome sequencing identified a homozygous canonical splice-site variant in *MCM10* (c.930 + 2T > A), resulting in an in-frame deletion of 17 amino acids within the oligonucleotide/oligosaccharide-binding (OB)-fold domain, confirmed at the RNA level.Immunophenotyping showed near-absent NK cells with increased NK-T cells and terminally differentiated CD8 T cells. While karyotype, telomere length, and radiation sensitivity were normal, limited MCM10 expression in replicating NK cells is suspected. During follow-up, the patient developed an aggressive Epstein–Barr virus–positive squamous cell carcinoma of the tongue, an exceedingly rare childhood malignancy.

**Conclusion:**

This case expands the phenotypic spectrum of *MCM10*-related inborn errors of immunity and supports a critical role for *MCM10* in NK-cell development and immune homeostasis. The occurrence of a virus-associated malignancy suggests susceptibility and impaired immune surveillance. Together with previously described defects in CMG-complex components, these findings are consistent with an emerging group of DNA replication–associated immune disorders.

**Supplementary Information:**

The online version contains supplementary material available at https://doi.org/10.1007/s10875-026-02045-7.

## Introduction

Accurate DNA replication during cell division is a crucial cellular process, relying on coordinated activity of various factors to ensure precision and timing. Disturbances in this highly regulated process can result in genomic instability, characterized by somatic accumulation of point mutations or chromosomal abnormalities [1]. There are about a dozen known congenital disorders caused by inherited defects in DNA replication genes [2]. These syndromes manifest with a wide variety of symptoms, some shared across all disorders, and others unique to a specific syndrome. The main common characteristic of these disorders is short stature, reflecting the crucial role of efficient DNA replication in overall growth. Other more specific manifestations, not shared by all syndromes, provide clues for the functions of each defective protein, and can help establish a clinical diagnosis. For example, Meier-Gorlin syndrome (MGORS), caused by defects in 13 genes (e.g. *ORC1*,* ORC4*,* ORC6*, *CDC45*) and known for a triad of short stature, microtia, and patellar abnormalities, is one of the first described syndromes caused by germline defects in DNA replication genes [3]. Other DNA-replication defects, such as Rothmund-Thomson syndrome type 2, Baller-Gerold syndrome and RAPADILINO syndrome, caused by pathogenic variants in *RECQL4*, are associated with increased risk of cancer, indicating that the RecQ DNA helicase family has a critical role in maintaining genomic stability [4].

The Minichromosome Maintenance (MCM) protein family, named after a 1984 study in which mitotically stable plasmids (‘minichromosomes’) were introduced into yeast in order to assess DNA replication and to identify genes essential for the replication process [5]. Upon initiation of the replication process, MCM2-7 function as the core of the replicative helicase, bind firing factors, including the go-ichi-ni-san (GINS) complex and the cell division cycle protein 45 (CDC45), to form the CDC45:MCM2-7:GINS (CMG) helicase complex [6]. MCM10, another member of the MCM family, facilitates the final priming of double-stranded DNA [7], and holds a vital role in stabilizing the CMG helicase complex, stimulating replication elongation and enhancing genome stability [8].

Although the significance of the CMG-complex proteins has become increasingly apparent in recent years, the occurrence of inborn errors of immunity (IEIs) associated with CMG-complex defects remains uncommon, with a limited number of cases described. As such, a rare autosomal recessive *MCM4*-deficiency was described in Irish travelers, causing immunodeficiency syndrome characterized by natural killer (NK)-cell deficiency, susceptibility to severe viral infections, short stature, and adrenal insufficiency [9, 10], and *GINS1*-deficiency (*go-ichi-ni-san* complex subunit 1) was associated with intrauterine growth retardation, NK-cell deficiency and neutropenia [11].

To date, pathogenic bi-allelic variants in *MCM10* were reported in two families. Mace et al. described a 16-month-old patient with NK-cell deficiency, who died of severe Cytomegalovirus (CMV) infection at the age of two years [12]. Baxley et al. described a recurrent prenatal phenotype in a single family, including fetal hydrops, restrictive cardiomyopathy, and hypoplastic thymus and spleen, resulting in intrauterine death or termination of pregnancy [13]. Accordingly, a new autosomal recessive IEI disorder was defined - immunodeficiency 80 with or without congenital cardiomyopathy (OMIM #619313).

Here, we describe a child with a novel homozygous *MCM10* splice-site variant and a severe immunologic phenotype characterized by NK-cell deficiency, expanding the clinical spectrum of *MCM10*-related inborn errors of immunity and highlighting a possible susceptibility to virus-associated malignancy.

## Methods

### Study Participant

A 6-year-old male, traveled to Israel to seek medical evaluation and was admitted to the Dana Dwek Children’s Hospital at the Tel Aviv Sourasky University Medical Center (TASUMC). He underwent comprehensive multi-disciplinary evaluations, including hematologic, dermatologic, genetic, immunologic, and otolaryngologic. Written informed consent was obtained.

### Genetic Analysis

We performed singleton exome sequencing in-house on an Illumina NovaSeq 6000 sequencer using the IDT xGen Exome Research Panel v2 for library preparation. The FASTQ files were uploaded to the Franklin by Qiagen (formerly Genoox) analysis platform, on which we performed the entire bioinformatic analysis, including alignment to the reference genome (GRCh37/hg19), variant calling, data filtering and candidate variant identification. Sequencing results were filtered for rare (gnomAD minor allele frequencing < 1%) protein-altering variants (missense, nonsense, indels, splice-site), and all inheritance patterns were considered during the analysis.

### RNA Studies

To study the effect of the candidate splice-site variant, we obtained the patient’s RNA using the PAXgene Blood RNA Kit on the QIAcube machine. cDNA was generated through reverse transcription with the qScript cDNA Synthesis Kit. Primers encompassing the candidate variant were used to observe potential splicing defects by gel electrophoresis and Sanger sequencing.

### Immune Evaluation and Flow Cytometry-Based Studies

Peripheral blood mononuclear cells (PBMCs) were isolated from fresh blood samples using Ficoll density gradient centrifugation and were stored in liquid nitrogen for later use.

For B and T cell immunophenotyping, and Double Negative T-cell evaluation, PBMCs were thawed and stained. Radiation sensitivity was evaluated using a previously described radiosensitivity flow assay [14]. Briefly, cells were exposed to low-dose (2 Gy) irradiation, and stained intracellularly for phospho-gH2AX at baseline, 1-hour post irradiation and at 24 h post irradiation.

Conjugated Fluorescence-Activated Cell Sorting (FACS) antibodies, as well as fixation and permeabilization reagents used in these experiments, are listed in Supplementary Table S1. Cells were acquired using a BD FACSCanto II flow cytometer and data analysis performed using FlowJo software (V10.0, TreeStar).

### Telomere Length Evaluation and Karyotyping

Clinical telomere length evaluation was performed at the Hematologic Laboratory, Schneider Children’s Medical Center, Petah-Tikva, Israel, using a Flow-fluorescence in situ hybridization (Flow-FISH) assay. Karyotype was completed at the Cytogenetic Laboratory, TASUMC, using standard protocols.

### Visualization in Single-cell RNA Sequencing (scRNA-seq) Dataset

*MCM10* expression across all types of PBMCs were evaluated using a publicly available scRNA-seq dataset, consisting of 149,564 cells aggregated into 23 cell types [15]. We used the “FeaturePlot” function in Seurat package (v3.1) to depict the relative expression of *MCM10* and various marker genes on a uniform manifold approximation and projection (UMAP) in single-cell resolution.

## Results

### Patient History

**Fig. 1:**
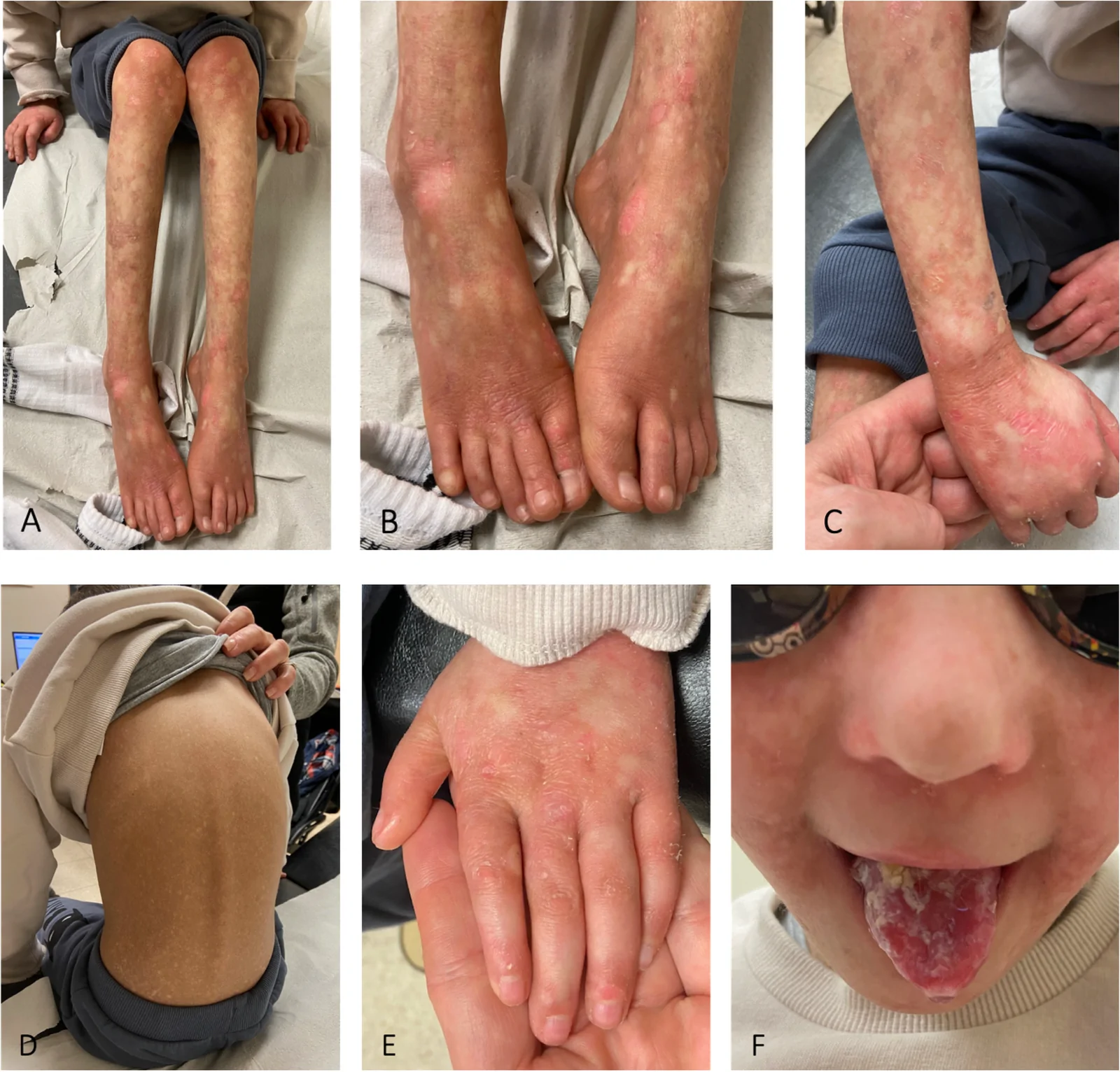
Skin eruption and oral plaques. Images show skin eruption with hypopigmented and hyperpigmented macules and patches, along with areas of flat erythematous papules covered with fine scales, involving the upper and lower extremities (**A**-**C**) and back (**D**). No significant onychosis or onychodystrophy was observed (**E**). Finally, white oral plaques, suspected to be oral candidiasis versus lichenoid lesions, were observed (**F**)

A 6-year-old male, an only child to healthy, non-consanguineous, Ukrainian parents, was born at term with a birthweight of 3100 g. He was reportedly healthy until the age of two years. Other than mild speech delay, motor and cognitive development were appropriate for age. At two years of age, he began developing a chronic skin eruption described as hypo- and hyperpigmented macules and patches, arranged in a reticular pattern, which gradually covered his entire skin surface. Additionally, some areas exhibited flat erythematous papules that were covered with fine scales (Fig. 1A-E). Hair and nails were spared. At 3.5 years, he developed eyelid lesions, which lead to several surgical interventions, resulting in chronic trichiasis and entropion with possible secondary symblepharon, scleral scarring and neovascularization of the right eye cornea. At the age of four, oral white plaques were noted and oral thrush versus lichenoid lesions were suspected (Fig. 1F). Biopsy of a single tongue lesion was reported to be compatible with a Candida fungal ball, although a formal report was not available, and improved oral hygiene or oral miconazole treatment resulted in limited and transient improvement only. Over the years, deceleration of growth was noted, and at age of five years, physical examination revealed failure to thrive (weight 14.5 kg, -3.0 SD) and microcephaly (head circumference 47 cm, -3.6 SD), with a relatively preserved height of 111 cm (8th percentile).

As for involvement of additional systems, occasional diarrhea was reported since the age of 5 years, with no formal gastroenterology evaluation, and echocardiography was normal with no evidence of cardiomyopathy. Regarding history of infections, he suffered a single episode of pneumonia around the age of 1.5 years, and other than the above-mentioned oral lesion, there was no history of significant viral or fungal infections.

Upon arrival to our care, the patient underwent repeated evaluation. Skin biopsy showed lichen simplex chronicus and dermal scar. Tongue biopsy showed epithelial hyperplasia, with significant hyperkeratosis, mild dysplastic changes and granulation tissue with heavy lympho-plasmocytic infiltration. Immunohistochemical stains for HPV, HSV1, HSV2 and CMV were all negative. Candida hyphae were not observed.

During follow-up, the patient developed an additional severe complication in the form of a rapidly progressive malignancy. A rapidly growing left sub-mandibular mass appeared. CT scan showed a dominant submental process and an extensive conglomerate of pathological lymph nodes in the left posterior neck and submandibular area. This time, repeated biopsies of the tongue ulcer and the cervical mass demonstrated squamous cell carcinoma (SCC) of the tongue with positive Epstein-Barr virus-encoded small RNA 1 (EBER1) staining, and involvement of all margins. Following the diagnosis of extended unresectable cancer with a poor prognosis, the family opted to return to Ukraine and the patient died shortly thereafter.

### Variant Identification and Splicing Defect Evaluation

Singleton exome sequencing identified a rare homozygous variant in *MCM10* (NM_018518.5): c.930 + 2T > A, with minor allele frequency of 0.001195% in gnomAD and no reported homozygous individuals. The variant was not observed in our internal database of ~ 20,000 exomes and genomes and was not described in the literature or reported in the ClinVar database.

**Fig. 2:**
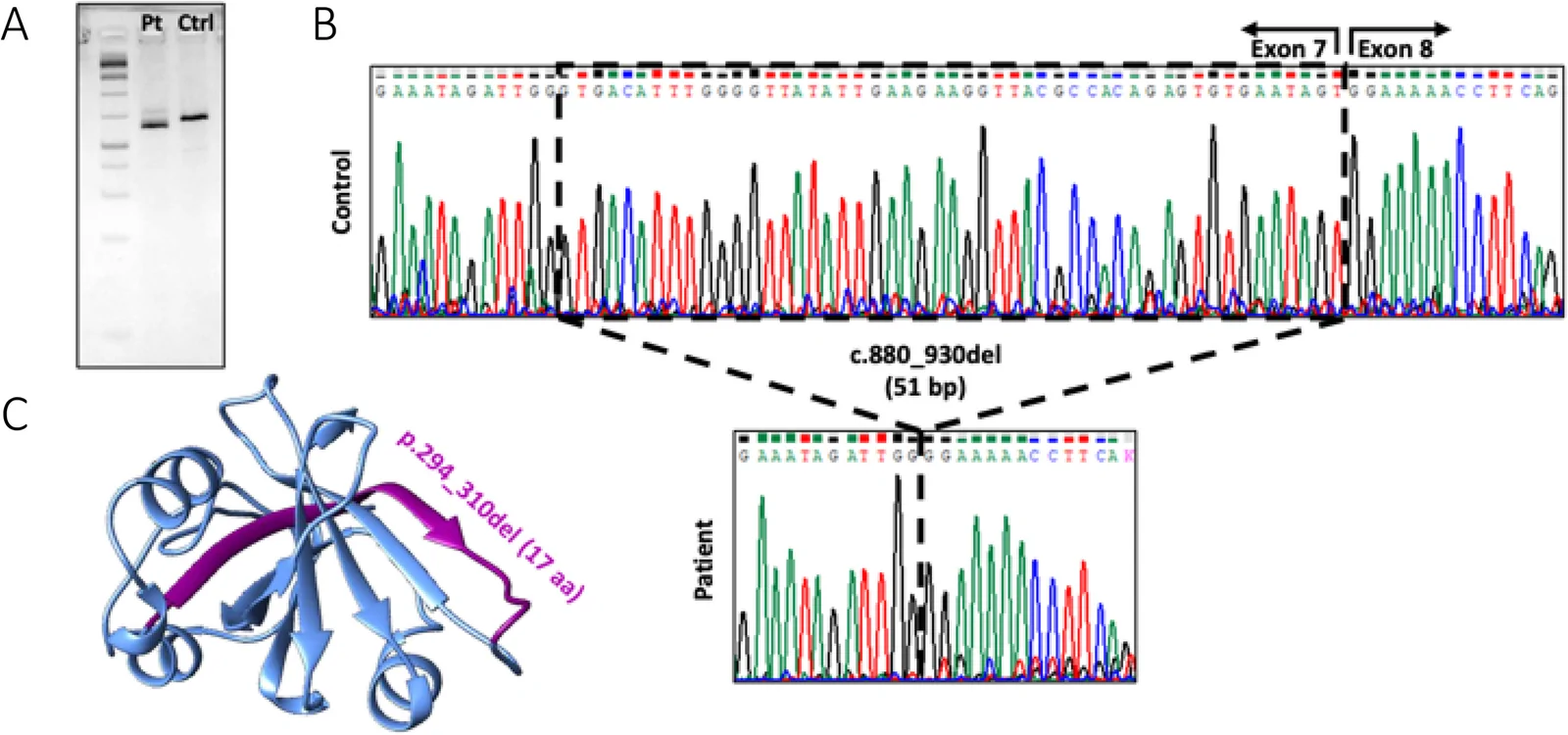
MCM10 splice-site variant. Agarose gel electrophoresis of amplified MCM10 cDNA, comparing patient and control samples, reveals a shorter MCM10 mRNA isoform in the patient (**A**). Sanger sequencing chromatograms of the MCM10 mRNA showing a homozygous in-frame deletion of 51 base pairs from the 3’-end of exon 7 (**B**). Three-dimensional model of the oligosaccharide-binding (OB)-fold domain of the MCM10 protein, highlighting (in purple) the 17 amino acids absent in the patient (**C**)

The c.930 + 2T > A variant was thought to affect the donor splice site of exon 7 (out of 20) of the *MCM10* gene and was expected to disrupt canonical splicing. Indeed, RNA analysis showed a shorter amplified *MCM10* mRNA isoform in patient RNA compared with an unrelated healthy control (parental samples were not available). Sequencing of the amplified region showed deletion of 51 bp from the 3’-end of exon 7, resulting in an in-frame deletion of 17 amino acids [n.880_930del; p.(294_310del)], located within the protein’s oligonucleotide/oligosaccharide-Binding (OB)-fold domain (Fig. 2A-C).

### Immune Evaluation

**Fig. 3:**
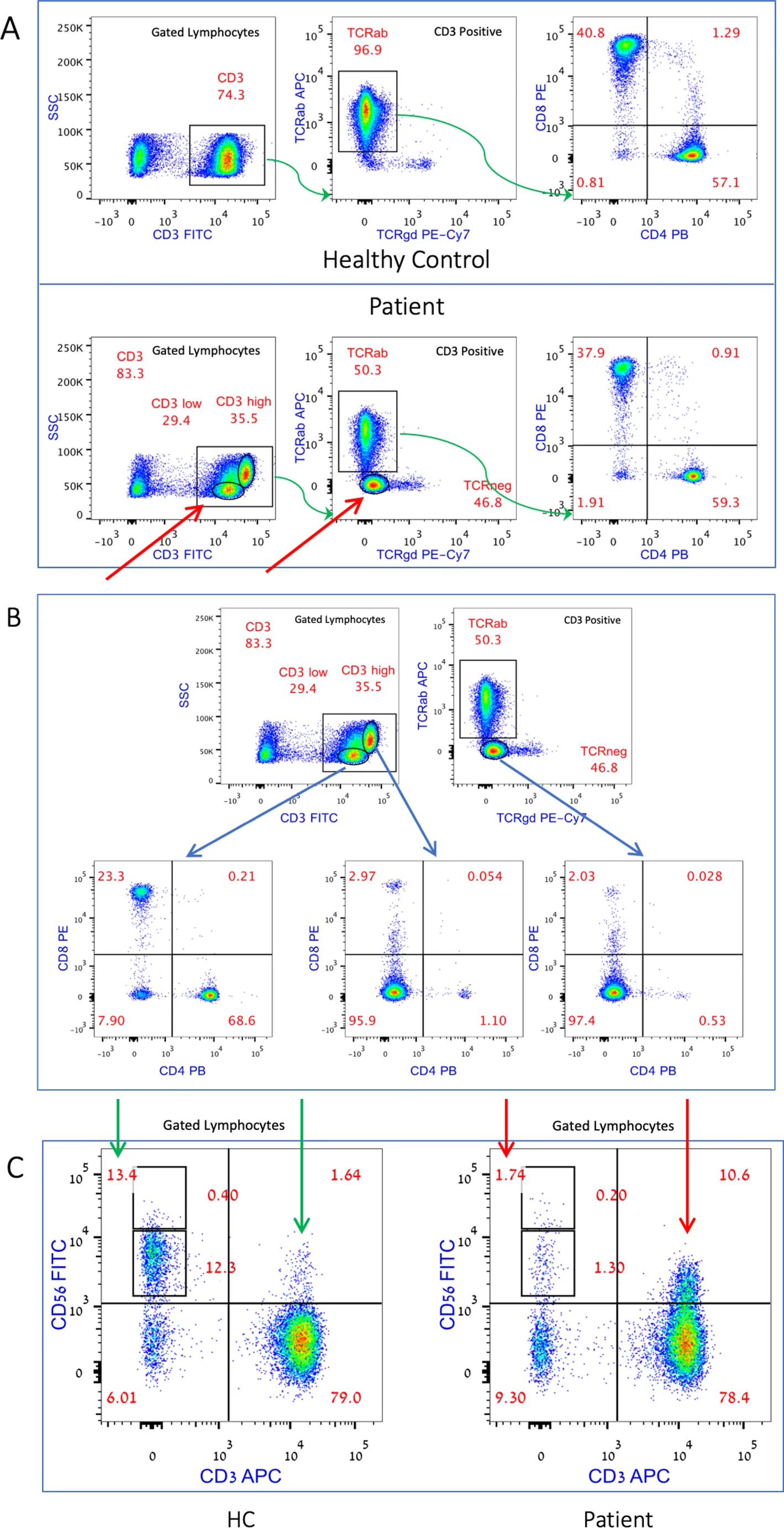
Flow cytometry-based immunophenotyping: ALPS screen and CD56 staining. While ALPS screen was normal with 1.9% of the CD3^+^TCRα/β^+^ being CD4/CD8 double negative cells (**A**), an unusual population of TCRαβ/TCRγδ negative CD3^+^ cells was observed, of which 95% were CD4/CD8 double negative and backgated to the CD3^high^ population (**B**). CD56 and CD3 staining showed low percentage of CD3^−^CD56^+^ cells, of which a significant relative percentage were CD56^bright^ cells (**C**). Green arrows in panel A mark the gating strategy

Initial immune evaluation showed normal IgG and IgA levels (946 mg/dL and 144 mg/dL, respectively), and mildly reduced IgM level (28 mg/dL). Lymphocyte subset analysis showed an absolute lymphocyte count of 2500/µL, with normal counts of CD3 (2028/µL), CD4 (750/µL), CD8 (425/µL) and CD19 (325/µL) cells, but low CD16/CD56 cells of 50/µL. Since the total number of CD4 and CD8 (1175/µL) did not correlate with the absolute CD3 lymphocyte count, an ALPS screen was preformed and showed two populations of CD3^+^ cells (Fig. 3A, lower left panel), including a large population of cells lacking both TCRαβ and TCRγδ expression (Fig. 3A, lower middle panel). These cells were 95% CD4/CD8 double negative and backgated to the CD3^high^ population (Fig. 3B). Of note, a similar discrepancy between the sum of the CD4 and CD8, and the total CD3, was also described by Mace EM et al. [12].

Additional evaluation showed abnormal B-cell immunophenotyping with low percentage and impaired class switching of the CD38^low^CD24^high^ memory B-cell population, and skewed T-cell immunophenotyping with an increased percentage of CD8 CD4RA^+^CCR7^−^ T_EMRA_ cells (Supplementary Figure S1A, B).

Following the identification of the novel *MCM10* homozygous variant, evaluation of the NK population was completed, revealing a low percentage of CD3^−^CD56^+^ cells, of which a significant relative percentage were CD56^bright^ cells (Fig. 3C), similar to the previously described in the MCM10-related patient [12]. Interestingly, a relatively high percentage of CD3^+^CD56^+^ cells was observed compared with control, which although not directly tested (due to limited amount of patient’s PBMCs), may correspond to the above-mentioned TCRαβ/TCRγδ negative CD3 population. The developmental origins and function of these aberrant lymphocytes remain to be determined.

### NK-deficiency is not Explained by Telomere Length or Radiation Sensitivity

In an attempt to better understand the mechanisms underlying the NK cell deficiency and the effects of an impaired MCM10 protein, additional tests were performed. Relative telomere length, evaluated by Flow-FISH assay, was within the normal range for age (Supplementary Figure S2A), inconsistent with a previous report showing reduced telomere length in cell lines with MCM10 deficiency [13]. Similarly, a peripheral blood karyotype was normal (Supplementary Figure S2B).

**Fig. 4:**
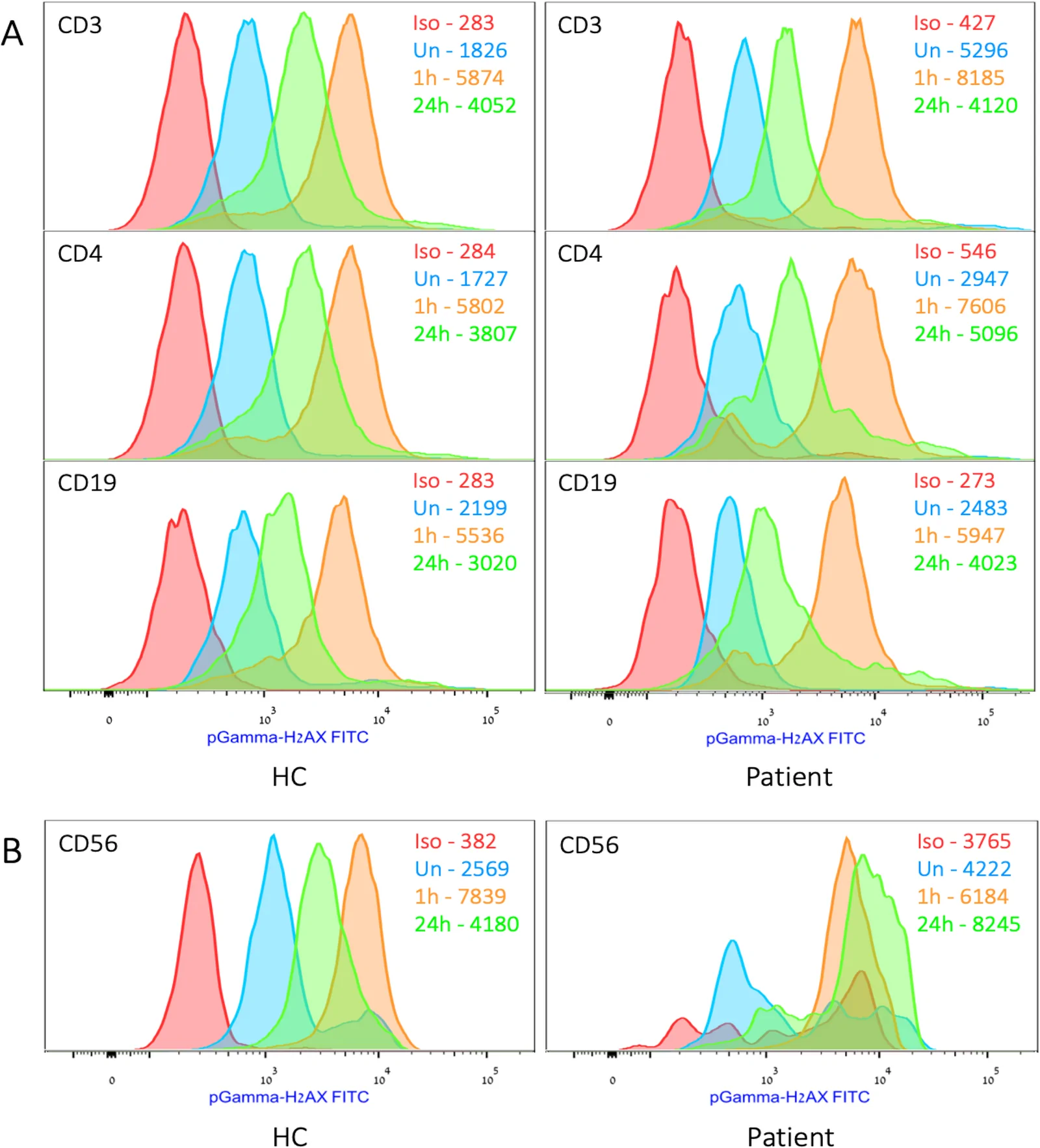
Pre- and Post-irradiation pγH2AX staining. Healthy Control (HC) and patient PBMCs were stained for pγH2AX at baseline, 1-hour post and 24 h post low-dose (2 Gy) irradiation. Mean fluorescent intensity (MFI) is shown for different cell populations. (**A**) comparable pγH2AX MFI was observed within the CD3, CD4 and CD19 populations in both control and patient samples. A higher pγH2AX MFI at 24 h post-irradiation was observed within the CD56^+^ population; however, interpretation is limited by the low number of recorded events in the patient sample (**B**). Red: Isotype control, Blue: Unirradiated, Orange: 1 h post irradiation, Green: 24 h post irradiation

Next, we tested patient’s primary cells for radiation sensitivity, using a pγH2AX flow assay. Irradiation of PBMCs with 2 Gy did not show an increase in pγH2AX MFI in either T cells or B cells at 1 and 24 h post irradiation (Fig. 4A), arguing against a general increase in radiation sensitivity or a general defect in DNA damage response. However, and despite the fact that limited cells availability did not allow proper analysis of pγH2AX staining within the CD56^+^ cell population, there was a tendency toward higher pγH2AX staining at 24 h in CD56^+^ cells (Fig. 4B), suggesting possible selective radiation sensitivity of MCM10-deficient NK cells, and specific role for MCM10 in NK cells.

**Fig. 5:**
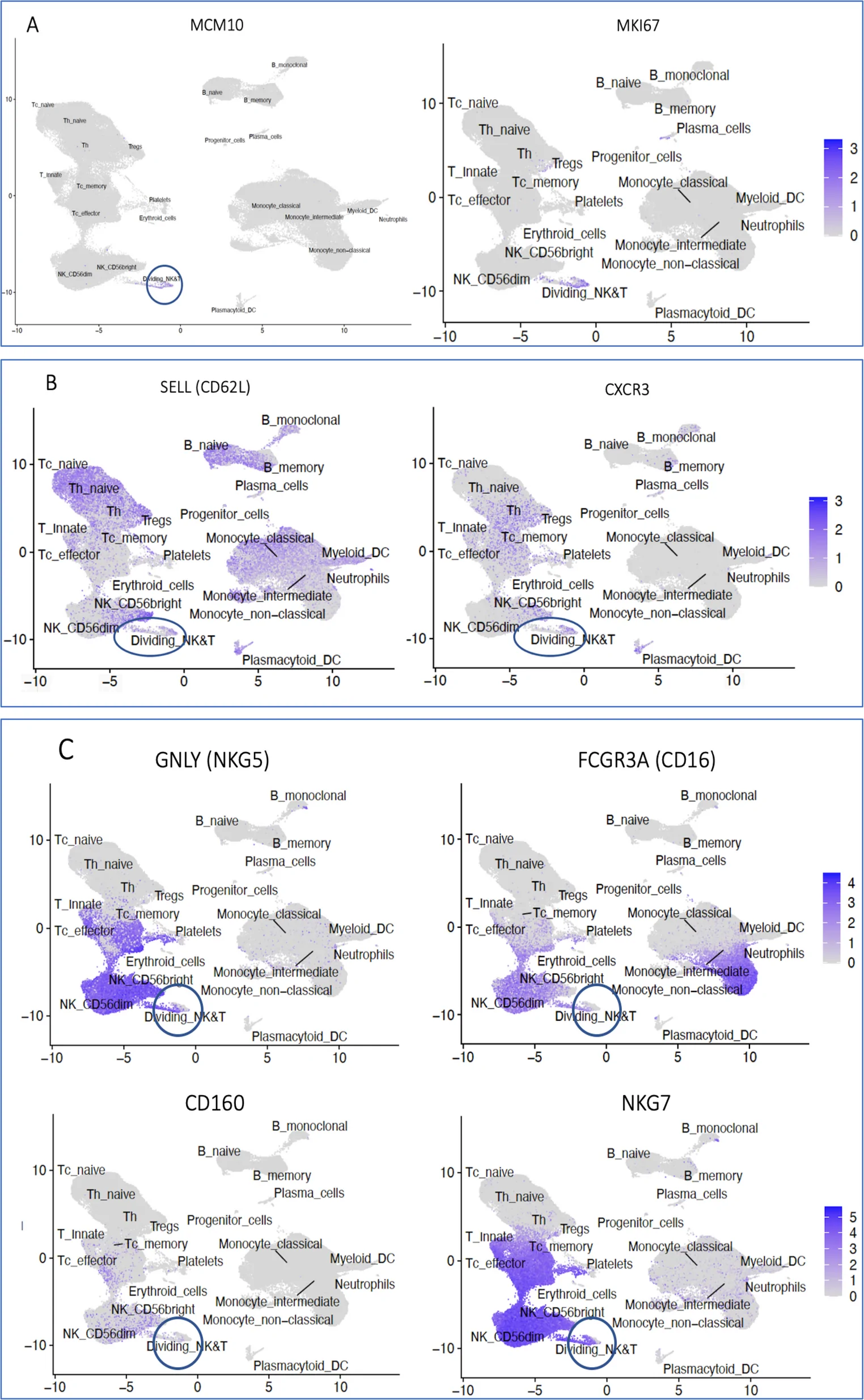

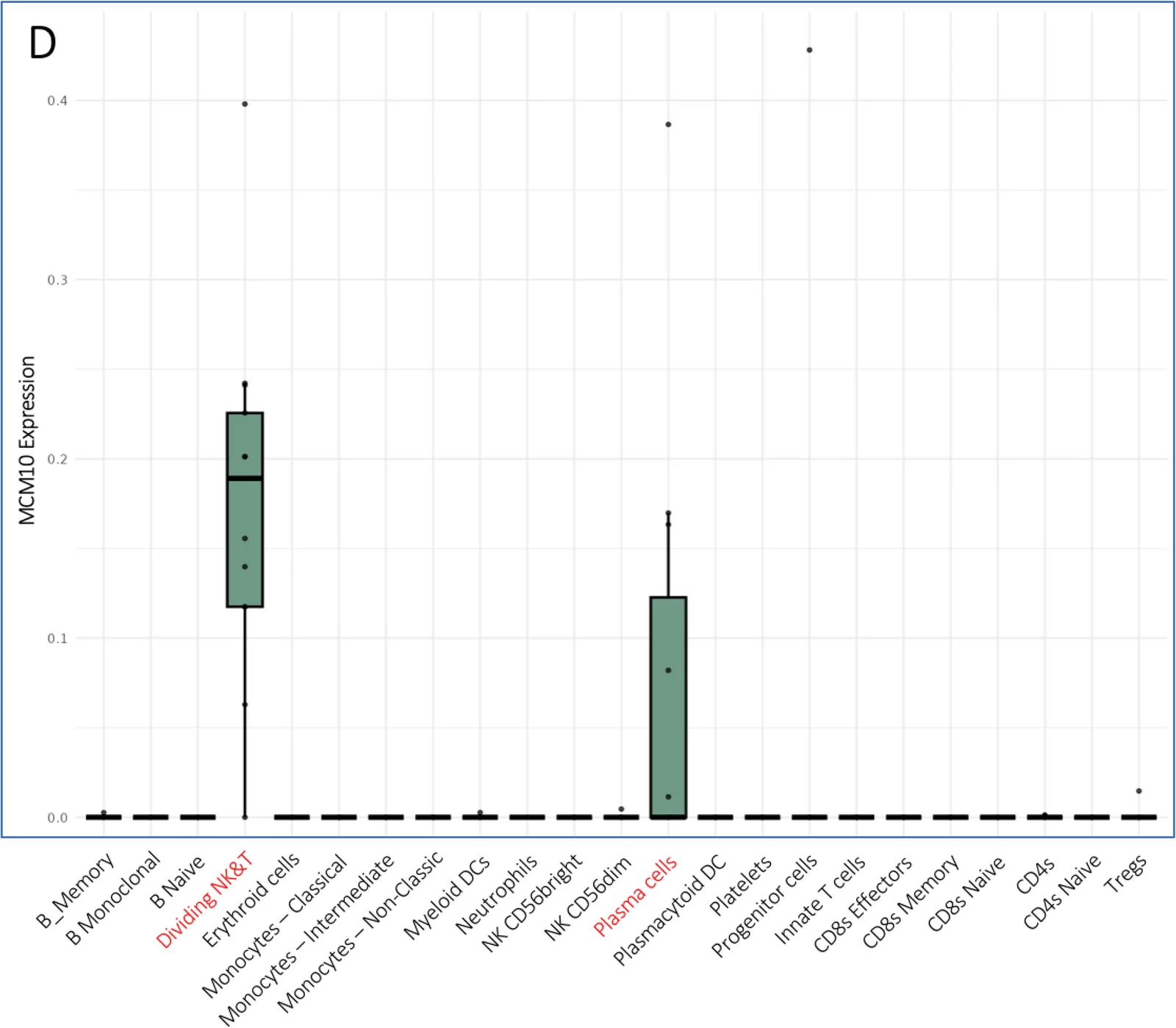
In-silico scRNA-seq data suggest overlapping expression of MCM10 with proliferating CD56^bright^-cell markers. ScRNA-seq data of PBMCs from a publicly available dataset was analyzed for the expression of MCM10 and different NK-cell markers. UMAP shows expression of MCM10 within proliferating NK cells and T cells, overlapping with KI67 expression (**A**). In addition, MCM10 also overlaps with the NK^bright^ markers, SELL (CD62L) and CXCR3 (**B**), and less so with NK^dim^ markers, FCGR3A (CD16), GNLY (NKG5), NKG7 and CD160 (**C**). Boxplot of the average expression in 13 healthy individuals better shows the differential expression of MCM10 in dividing NK and T cells, as well as plasma cells (**D**)

While additional PBMCs were not available for further studies, evaluation of publicly available PBMC scRNA-seq data [15] demonstrated that *MCM10* expression is limited mostly to proliferating (MKI67⁺) NK and T cells (Fig. 5A and D), overlapping with markers of CD56^bright^ NK cells (SELL, CXCR3) (Fig. 5B), and less so with markers of CD56^dim^ NK cells (*FCGR3A* [CD16], *GNLY* [NKG5], *NKG7* and *CD160*) (Fig. 5C). This pattern of RNA expression could suggest that MCM10 is essential for early development of NK cells, and lack of its proper expression leads to NK-deficiency with relative predominance of CD56^bright^ cells. Complementary analysis of mini-bulk RNA-seq data (ImmGen Databrowsers, https://rstats.immgen.org/Skyline_human/ICA_Skyline.php) further supported differential *MCM10* expression across immune subsets, showing that *MCM10* expression is upregulated between CD56^bright^ immature and CD56^dim^ mature NK subsets (Supplementary Fig. 3). If that is indeed the case, we could expect MCM10 deficiency to affect mature CD56^dim^ NK cells, while sparing CD56^bright^ cells.

## Discussion

We report a child with biallelic *MCM10* deficiency presenting with NK-cell deficiency, immune dysregulation, and cutaneous manifestations, thereby expanding the phenotypic spectrum of *MCM10*-related inborn errors of immunity. In addition, the patient developed an aggressive EBV-positive squamous cell carcinoma of the tongue, an extremely rare malignancy in childhood, which may reflect increased susceptibility to virus- associated disease in the setting of impaired immune surveillance.

Germline pathogenic variants in *MCM10* were first described several years ago, causing NK-cell deficiency with lethal cytomegalovirus infection or, prenatally, cardiomyopathy with hypoplastic thymus and spleen and fetal hydrops [12, 13]. To date, only four affected individuals from two families with biallelic *MCM10* variants have been reported, all carrying one loss-of-function and one hypomorphic allele [13].

Experimental models and human studies collectively support a critical role for *MCM10* in embryonic development, immune development and genome stability. *Mcm10* knockout mouse embryos fail early in development [16], while zebrafish models show impaired hematopoietic stem cell emergence with increased DNA damage and apoptosis [17]. In human cell lines, reduced MCM10 levels lead to replication fork slowing and increased DNA damage at common fragile sites, supporting a role for *MCM10* in safeguarding genome integrity [18].

Accordingly, functional studies in patient-derived cells with biallelic *MCM10* variants have demonstrated increased replication stress, impaired cell-cycle progression, and defective NK-cell maturation, consistent with the NK-cell deficiency observed in affected individuals [12]. Additional work has linked *MCM10* deficiency to chromosomal instability and telomere shortening [13]. More recently, induced pluripotent stem cell models showed that reduced MCM10 dosage selectively impairs differentiation into mature NK cells, with minimal effects on other lymphoid lineages, suggesting a unique NK-cell–specific vulnerability [19].

In this study, we present a patient with a pathogenic homozygous splice-site variant located in intron 7 of the *MCM10* gene. We demonstrate the causative nature of this variant, which leads to a deletion of 51 bp, causing an in-frame deletion of 17 amino acids, located within the protein’s oligonucleotide/oligosaccharide-Binding (OB)-fold domain. The OB-fold domain is a major ssDNA/RNA-binding domain, structured as a five-stranded closed β-sheet barrel, and is critical for MCM10 function through its role in DNA synthesis re-initiation and genome integrity preservation [20]. The c.930 + 2T > A variant leads to deletion of amino acids 294 through 310, which make up one of the domain’s β-sheets. Although we were not able to test MCM10 expression at the protein level in patient’s cells, it is expected that this deletion would either interfere with normal MCM10 function or cause protein instability and degradation.

While it is reasonable to assume that replication dysregulation and impaired genomic stability would explain NK-cell deficiency, it is still unclear why such defects would affect NK cells specifically, without affecting other hematopoietic cells. In contrast to previous reports, we did not identify abnormalities in karyotype or telomere length, nor did we observe radiation sensitivity. However, in accordance with Schmit et al. [19], who showed unique effect of heterozygous *MCM10*-loss on the ability of iPSCs to differentiate into mature NK cells, our analysis of available PBMC scRNAseq data, supports a stage-specific requirement for MCM10 during NK-cell maturation, and suggests selective and differential *MCM10* RNA expression within proliferating NK cells, and specifically in those expressing markers of CD56^bright^ NK cells. This pattern of expression would correlate with the observed predominant CD56^dim^ deficiency.

Importantly, these findings place MCM10 within a growing group of DNA replication–associated proteins whose partial loss preferentially disrupts immune development, particularly affecting natural killer cell maturation. Similar immunologic phenotypes have been described in individuals with defects in other components of the CMG helicase complex, suggesting a shared pathogenic mechanism. Taken together, inherited defects in components of the CDC45–MCM2-7–GINS (CMG) helicase complex and its associated factors, including MCM4, GINS1, and MCM10, define a small but emerging group of inborn errors of immunity caused by impaired DNA replication machinery. We propose referring to this group collectively as **“minichromosomopathies**,**”** reflecting their shared molecular basis in defective minichromosome maintenance and DNA replication. Further delineation of this proposed group will require additional patients and functional studies across different tissues.

Earlier studies focused on the effect of MCM10 on hematopoietic cells in general, and on NK cell development specifically. However, the severe skin disorder observed in our patient could provide insights into the essential role of MCM10 in preserving genomic stability and providing protection against replication stress in other non-hematopoietic tissues. Similarly, disrupted regulation of cell replication resulting from aberrant MCM10 function could offer a plausible explanation for our patient’s additional features, including notable microcephaly and failure to thrive.

SCC of the tongue is an extremely rare cancer in children [21, 22], as the conventional risk factors include smoking, alcohol consumption, tobacco chewing, and long-standing human papillomavirus (HPV) infection. A relationship between head and neck SCC development in children and known genetic syndromes has been previously described, e.g. Fanconi’s anemia, Bloom’s syndrome, Xeroderma Pigmentosa and Dyskeratosis Congenita, among others [23, 24]. These associations imply that genomic instability or chronic inflammation play a critical role in the development of oral cancers [25]. In addition, EBV-positive SCC, by itself a rare finding, could suggest EBV-susceptibility, and may imply the presence of an underlying IEI [26, 27]. Therefore, the development of EBV-positive SCC in our patient may reflect the combined effects of genomic instability, NK-cell deficiency with impaired ability to clear EBV-infected epithelial cells, and a potential role of MCM10 in epithelial tissue. While this observation raises concern for malignancy susceptibility in MCM10 deficiency, conclusions regarding cancer predisposition are limited by the single-patient nature of this report.

In conclusion, this case expands the clinical spectrum of *MCM10*-related disease and supports a critical role for *MCM10* in NK-cell development and immune homeostasis. The occurrence of an EBV-positive carcinoma in this patient suggests that *MCM10* deficiency may confer susceptibility to virus-associated malignancy, a possibility that warrants further investigation in additional patients. Together with previously reported defects in CMG-complex components, these findings support the emerging concept of **“minichromosomopathies**,**”** characterized by immune dysregulation and, potentially, increased vulnerability to malignancy.

## Supplementary Information

Below is the link to the electronic supplementary material.

Supplementary Material 1 (DOCX 18.2 MB)

## Data Availability

No datasets were generated or analysed during the current study.
