# Supplementary material for: Biallelic MCM10 Deficiency Presenting with Natural Killer Cell Deficiency, Poikiloderma and EBV-Associated Malignancy
Source: J Clin Immunol. 2026 Jul 30;46(1):98. doi: 10.1007/s10875-026-02045-7 (PMC13710629; doi:10.1007/s10875-026-02045-7)
Supplement: Supplementary file 1 [file 10875_2026_2045_MOESM1_ESM.docx]

**Biallelic MCM10 Deficiency Presenting with Natural Killer Cell Deficiency, Poikiloderma and EBV-Associated Malignancy**

Yael Hoffman MD^1,4†^, Alina Kurolap RN PhD^1^, Hila Ledermann Nahmias PhD^1^, Adi Mory PhD^1^, Tal Freund MSc^2^, Avraham Unterman MD MBA^3,4^, Uri Hamiel MD^1,4^, Hagit Baris Feldman MD^1,4^, Daphna Marom MD^1,4,*^, David Hagin MD PhD^2,4,*,†^

**Supplemental Table and Figures**

**Table S1: Antibody panel used for flow cytometry.**

| **Panel** | **Target** | **Clone** | | **Fluorophore** | **Manufacturer** |
| --- | --- | --- | --- | --- | --- |
| **B-cell Memory Panel** | CD19 | HIB19 | | PE/CY7 | Biolegend |
|  | CD27 | M-T271 | | APC/CY7 | Biolegend |
|  | IgM | MHM-88 | | Pacific Blue | Biolegend |
|  | IgA | Goat anti-human | | FITC | Southern Biotech |
|  | IgG | M1310G05 | | BV510 | Biolegend |
|  | BAFF-Receptor | 11C1 | | PE | Biolegend |
|  | | | | | |
| **B-cell Maturation panel** | CD19 | HIB19 | | PE/CY7 | Biolegend |
|  | CD27 | M-T271 | | APC/CY7 | Biolegend |
|  | CD38 | HIT2 | | PerCP/Cy5.5 | Biolegend |
|  | IgM | MHM-88 | | FITC | Biolegend |
|  | IgD | IA6-2 | | BV510 | Biolegend |
|  | CD10 | HI10a | | APC/CY7 | Biolegend |
|  | CD24 | ML5 | | PE | Biolegend |
|  | CD21 | HB5 | | Super Bright 436 | eBioscience™ |
|  | | | | | |
| **T-cell** | CD4 | RPA-T4M | | Pacific Blue | Biolegend |
|  | CD8a | RPA-T8 | | PE | Biolegend |
|  | CD45RA | HI100 | | FITC | Biolegend |
|  | CD45RO | UCHL1 | | APC/CY7 | Biolegend |
|  | HLA-DR | L243 | | PE/CY7 | Biolegend |
|  | CCR7 | G043H7 | | AF®647 | Biolegend |
|  | | | | | |
| **ALPS screen and NK staining** | CD3 | | UCHT1 | FITC | Biolegend |
|  | CD4 | | RPA-T4M | Pacific Blue | Biolegend |
|  | CD8a | | RPA-T8 | PE | Biolegend |
|  | CD56 | | MY31 | PE | BD |
|  | TCRab | | IP26 | APC | Biolegend |
|  | TCRgd | | B1 | PE/CY7 | Biolegend |
|  | | | | | |
| **pγH2AX** | CD4 | RPA-T4M | | Pacific Blue | Biolegend |
|  | CD3 | OKT3 | | APC | Biolegend |
|  | CD19 | HIB19 | | PE/CY7 | Biolegend |
|  | CD56 | MY31 | | PE | BD |
|  | H2AX pS139 | REA502 | | FITC | Miltenyi Biotec |
|  | Human IgG1 Isotype Control | REA293 | | FITC | Miltenyi Biotec |

***Figure S1: B and T cell Immunophenotyping.*** B-cell immunophenotyping demonstrates abnormal B-cell immunophenotyping with low percent and impaired class switching of the CD38^low^CD24^high^ memory B-cell population (A). T-cell immunophenotyping shows skewing of CD8^+^ T-cell maturation with a reduced proportion of CD45RA^-^CCR7^+^ central memory T-cells (T_CM_), and an increased proportion of CD8 CD45RA^+^CCR7^-^ T_EMRA_ cells (B).

***Figure S2: Telomere length and Karyotype.*** Flow-FISH assay of telomere length showed normal relative age-matched mean length (red( (A). Karyotype analysis showed a normal chromosomal complement (representative image is shown in B).

***Figure S3: Public Data Mini-Bulk RNAseq.*** Publicly available mini-bulk RNAseq data (ImmGen Databrowsers, <https://rstats.immgen.org/Skyline_human/ICA_Skyline.php>), shows differential MCM10 expression across immune subsets, with higher expression in CD56^dim^ cells, as well as in activated Tregs and conventional type-I dendritic cells (DC1).
